# Reasoning decline during aging under familiar and unfamiliar physics

**DOI:** 10.3389/fragi.2025.1646655

**Published:** 2025-12-16

**Authors:** Melissa Dexter, Hélène Grandchamp des Raux, Ori Ossmy

**Affiliations:** 1 Centre for Brain and Cognitive Development, Birkbeck, University of London, London, United Kingdom; 2 School of Psychological Sciences, Birkbeck, University of London, London, United Kingdom; 3 Centre for Educational Neuroscience, Birkbeck, University of London, London, United Kingdom

**Keywords:** physical cognition, reasoning, aging, altered gravity, adaptability, flexibility

## Abstract

Physical reasoning is the capacity to anticipate how an environment will change as its elements move and interact. This cognitive skill, which is based on humans’ intuitive knowledge of physics, underlies everyday tasks that are potentially critical to older adults, such as avoiding collisions. Nevertheless, the effects of aging on physical reasoning remain understudied. Here, we tested physical reasoning among younger (18–35 years) and older (over 65 years) adults as they completed different difficulty levels of a physical reasoning paradigm. Participants viewed object displacements in a virtual environment and had to decide the outcome of that displacement under different gravity forces (terrestrial gravity, half, and double terrestrial gravity). We also tested distinct physical action concepts—supporting, launching, and clearing—because those index different demands on object-interaction complexity and are known to differ during child development. This allowed us to determine whether age-related differences reflect a global decline in physical reasoning or a selective difficulty with conceptually more complex, multi-object predictions. Our results revealed that older adults performed comparably to younger adults in straightforward *fail* conditions but exhibited lower accuracy in more complex scenarios, implicating subtle object interactions and predicting successful outcomes. This decline did not intensify under altered gravity, suggesting that the ability to recalibrate to new physical contexts may not be selectively affected by aging. However, older adults were disproportionately challenged by tasks featuring action concepts involving more complex object interactions, indicating that higher complexity burdens physical reasoning in later life. These findings highlight how intuitive physics can still degrade in key aspects of precision and complexity. Understanding these shifts is important for developing supportive strategies that help maintain functional independence in older adulthood, particularly in tasks requiring challenging physical reasoning.

## 1 Introduction

### 1.1 Physical reasoning

Humans’ interactions with the physical world are profoundly shaped by their ability to perceive, understand, and predict the outcomes of physical events (Fischer et al., 2016; Pramod et al., 2022). Even the simplest daily activities, such as pouring a drink or stacking household items, harness cognitive resources that enable individuals to anticipate and respond to local changes in their physical environment (Fischer et al., 2016; Mitko and Fischer, 2020). This predictive skill is grounded in prior perceptual experiences that allow the observer to simulate future scenarios by considering the properties of objects, such as mass, density, and friction, as well as their spatial configuration and relationships to one another (Baradel et al., 2019; Battaglia et al., 2013; Kubricht et al., 2017; Léoni et al., 2002; Vicovaro, 2023).

Reasoning about physical events relies on internal representations of physical principles that support inferences beyond immediate perception (Fischer et al., 2016; Vicovaro, 2023). Contemporary accounts formalise these representations as probabilistic “physics engines“ that continuously update beliefs about complex scenes (McCloskey, 1983; Battaglia et al., 2013; Kubricht et al., 2017; Smith et al., 2013; Ullman et al., 2017). In Bayesian terms, observers combine priors—rooted in an internalised understanding of Newtonian regularities—with current sensory evidence to forecast causal outcomes, even under perceptual noise (Battaglia et al., 2013; Mrowca et al., 2018; Vicovaro, 2023; Allen et al., 2020; Baradel et al., 2019; Kubricht et al., 2017). These predictive computations are tightly coupled to action planning, which must compensate for visuomotor delays of ∼150–200 ms by anticipating future states (Zago et al., 2008; Russo et al., 2017). When viewing time permits, internal models are refined and reasoning improve; when sensory input is sparse, people fall back on “good-enough“ Newtonian heuristics—illustrated in studies of ball motion where combined visual evidence and internal simulation yield the most accurate forecasts (Russo et al., 2017; Smith et al., 2013; La Scaleia et al., 2014).

### 1.2 Changes in physical reasoning with aging

Can physical reasoning change with time? Previous research addressed this question by predominantly focusing on the first years of life only. Indeed, findings in developmental science suggest that the development of physical reasoning is linked to the emergence of intuitive knowledge about physics, various cognitive capabilities, and motor development (Ossmy et al., 2020; Ossmy et al., 2022; Adolph and Robinson, 2015; Grandchamp des Raux et al., 2024; Baillargeon, 1995; Spelke and Kinzler, 2007; Allen et al., 2024). Thus, physical reasoning seems to be plastic and organised by action concepts—like ‘supporting,’ ‘launching,’ and ‘clearing’ (Grandchamp des Raux et al., 2024)—that capture how objects typically interact, and they get more complex as they involve coordinating more objects and movements.

Much less is known about whether and how physical reasoning diminish with age. Older adults have more difficulties in making inverse judgments about relationships between object mass, volume, and density compared to young adults. This may be attributable to an overreliance on experience-based heuristics (Léoni et al., 2002), but no direct evidence has provided such a link, despite older adults also experiencing declines in visual imagery and processing, which may be useful for physical reasoning (Craik and Dirkx, 1992). Aging also affects the ability to process allocentric spatial references, leading to difficulties in maintaining an accurate representation of spatial relationships among objects in one’s environment (Ruggiero et al., 2022; Salthouse and Mitchell, 1989). Such deficits interfere with older adults’ capacity to form precise predictions about physical interactions (Vicovaro, 2023). These changes are not uniformly experienced across all individuals or spatial skills (Iachini et al., 2009), and are dictated by the specific demands of the task (Iachini et al., 2009).

Moreover, older adults exhibit impairments in attentional control, particularly in the “alerting“ function, where they derive less benefit from environmental cues that facilitate predictive accuracy (Veríssimo et al., 2022). Such cognitive systems, which may be involved in physical reasoning, appear to be heavily reliant on early visual processing that occurs before higher-order cognitive processing begins, implying that effective prediction of physical events depends not only on the generation of spatial representations but also on the capacity to sustain attention and inhibit distractors effectively (Vicovaro, 2023; Wong et al., 2023). This is particularly crucial for older adults, who may be more susceptible to missing important cues from their surroundings.

Finally, previous research is limited in testing whether and how aging influences the generalization of physical reasoning across novel contexts (Pramod et al., 2022). Humans can refine the internal representation of the physical world through experience (Mitko and Fischer, 2020) and flexibly apply existing knowledge about physics to new contexts, such as worlds with altered physical laws or degrees of gravity (Battaglia et al., 2013; Kubricht et al., 2017; Russo et al., 2017). Addressing these issues is of growing importance as society strives to understand how the cumulative effect of environmental exposures, lifestyle choices, and socioeconomic disparities can lead to differential aging trajectories (Hancock and Ossmy, 2024; Kuh et al., 2014; C. Wang et al., 2021) and to mitigate inequalities experienced by older adults, particularly in complex urban environments where physical-reasoning abilities are essential for maintaining safety and autonomy.

### 1.3 Current study

In the present study we examined the aging of physical reasoning and adaptation of physical reasoning (Kubricht et al., 2017; Ullman et al., 2017) by testing the differences in physical prediction between older and younger adults. Specifically, we tested young adults (18–35-year-olds) and older adults (over 65 years) in a series of online physical reasoning games based on the Virtual Tools framework (Allen et al., 2020; Grandchamp des Raux et al., 2024). Based on previous aging research showing decline of cognitive processes such as spatial attention, working memory, and probabilistic reasoning (Berg et al., 1982; Iachini et al., 2009)—critical components underlying physics understanding and reasoning—we hypothesized that (H1) younger adults would triumph older adults in physical reasoning.

Moreover, we are not aware of studies that assess whether aging alters adaptability to unfamiliar physics. Considering research suggesting the use of priors when making future predictions (Battaglia et al., 2013), processing delays in older adults (Craik and Dirkx, 1992), and an increased exposure to the physical world by virtue of age, it is possible that older adults may rely more on priors when making predictions about future outcomes (Salthouse and Mitchell, 1989; Iachini et al., 2009; Veríssimo et al., 2022). Given the importance of reasoning adaptability, we also sought to test age differences in reasoning adaptability by assessing how participants perform when required to adapt their reasoning to altered physical laws, specifically different levels of gravity. Gravity is a force everyone experiences, regardless of age, and using different levels of gravity requires participants to adapt their physical knowledge and make physical predictions. We hypothesized that (H2) older adults would perform worse when required to adapt their reasoning. Adaptability was tested in trials using altered gravities.

Finally, we tested distinct physical action concepts—supporting, launching, and clearing—because those index different demands on object-interaction complexity and are known to differ during child development (Grandchamp des Raux et al., 2024). This allowed us to determine whether age-related differences reflect a global decline in physical reasoning or a selective difficulty with conceptually demanding, multi-object predictions. We hypothesized that (H3) ageing would affect action concept differently, similar to how development affects children. That is, the strongest effects of ageing on the Clearing concept, intermediate for Launching, and weakest for Supporting.

## 2 Methods

### 2.1 Participants

A total of 80 adults were recruited across two groups: 40 younger adults (YA, M = 30.35 years, SD = 6.03; 20 females) and 40 older adults (OA, M = 70.15 years, SD = 5.08; 20 females). One participant from the YA group was excluded due to technical issues in recording the data. All participants were United Kingdom or US residents. Our only inclusion criteria for each group were age (18–45 years of age for the YA group; 65 and older for the OA group), ability to use simple computer functionalities such as pressing a button for Yes or No, and no presence of long-term physical or neurological conditions. Given our focus on physical reasoning rather than motor execution, we adopted broad age-based inclusion criteria and we did not have any further exclusion criteria. Task demands were minimal (mouse click; no time limits). To ensure comprehension/engagement equivalence across groups, we used Easy-Fail trials as an *a priori* attention/engagement screen; Easy-Fail accuracy was high and statistically comparable across age groups.

Adults were recruited in Prolific—an online recruitment website. All participants were provided with a payment of £5 via the Prolific platform following the completion of the experiment for taking part. The study was granted ethical approval by the Psychology Ethics Committee at Birkbeck, University of London (Reference # 2122022).

### 2.2 Design and materials

**FIGURE 1:**
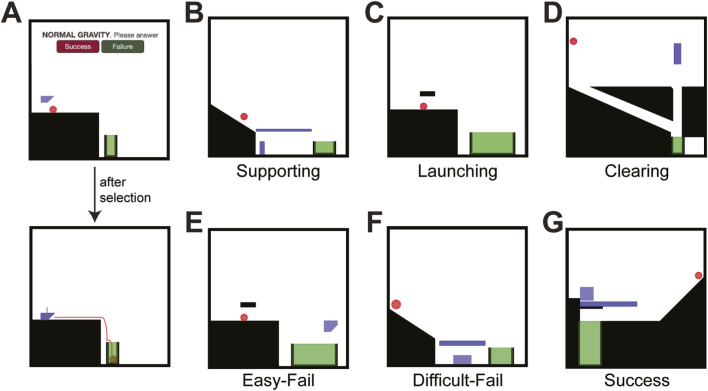
Prediction game. **(A)** Illustrative trial of the prediction game. Participants were required to determine whether the pre-placed blue tool in the specific location would lead to a cascade of physical events that would bring the red ball to the green area. Once they clicked on either the success (red ball gets to the target) or failure (red ball does not get to the target) button, the laws of physics started and showed the participants the outcome. **(B–D)** Exemplar games for the Supporting, Launching, and Clearing action concepts, respectively. **(E)** Exemplar Easy-Fail trial in which the tool was placed in a location that clearly led to a failure. **(F)** Exemplar Difficult-Fail trial in which the tool was placed in a location that causes a failure but the outcome is not obvious. **(G)** Exemplar Success trial in which the square tool was placed in a location that leads to a successful outcome. In this and some other levels, there were additional blue objects existing in the environment and these would add complexity to the prediction needs of the level.

The experiment adapts reasoning games from a gaming package called Virtual Tools, introduced by Allen et al. (2020). This package was previously used to assess physical reasoning across different age groups (for the original game levels, see https://k-r-allen.github.io/tool-games/). In the original games, participants are presented with dynamic two-dimensional virtual environments containing various virtual objects and shaded areas and are required to select and place a ‘tool’ to bring an object into a goal area. Upon placing the tool in the scene, the static environment starts to move per the laws of physics (e.g., gravity and collision forces). However, unlike the original games (Allen et al., 2020; Allen et al., 2024; Grandchamp des Raux et al., 2025), participants were not asked to select and place “tools“ themselves. Instead, they observed a pre-determined tool placement and were asked to predict whether the action would successfully achieve the goal—bring the red object to the green area (Figure 1A). This simplified interaction focuses specifically on outcome prediction, requiring participants to reason about the physical interactions within the virtual environment without manipulating the tools directly. Therefore, our measure of performance is solely based on the accuracy of their predictions (right or wrong), rather than the more complex metrics used in prior Virtual Tools research (e.g., number of attempts, timing, tool selection, placement). This distinction is crucial, as it isolates the predictive aspect of physical reasoning from the motor planning and execution components involved in actively manipulating the virtual tools.

We used 23 games from Virtual Tools, which differed in their physical action concepts (see Supplementary Table S1 for the list of game and their mapping to physical action concepts; Grandchamp des Raux et al., 2024), allowing us to test our third hypothesis regarding the role of action concepts in the potential decline of reasoning during aging. Physical action concepts represent the stored knowledge of how actions operate in the physical world (Allen et al., 2024; Leshinskaya et al., 2020). Similar to previous work (Grandchamp des Raux et al., 2024), our games included three such concepts—supporting, launching, and clearing—that vary in both motor complexity and cognitive demands for predicting physical outcomes. The first, supporting, entails sustaining an object’s structural integrity, balance, and weight distribution to keep it in a goal position (Figure 1B; for more details on physical action concepts and Virtual Tools see Grandchamp des Raux et al., 2024). The second, launching, involves propelling an object along a controlled trajectory and requires more refined coordination, as illustrated by hitting a stationary ball with a golf club (Figure 1C). Finally, clearing refers to removing or relocating obstacles from a given area (Figure 1D), a process that demands particularly advanced motor skills and the ability to anticipate and coordinate multiple object movements (e.g., clearing debris from a pathway). Importantly, in line with Grandchamp des Raux et al., 2024, we define concept difficulty by the interactional structure of the scene (number/coordination of object motions), so clearing remains most demanding even after motor placement was removed.

Participants observed three trial types for each game: (1) Easy-Fail—the tool was placed in a location that clearly led to a failure in which the red object could not reach the green area (Figure 1E; the tool placement was in a different part of the environment and so it was clear that it would not interact with either the red object or any existing items in the scene); (2) Difficult-Fail—the tool was placed in a location that causes a failure (red shape would not reach the goal area) but the outcome is not obvious (Figure 1F); (3) Success—the tool was placed in a location that leads to a successful outcome where the red shape reaches the goal area (Figure 1G). Success trial types varied significantly in difficulty, including both easier and more difficult scenarios to predict. Accuracy was measured by counting correct (Success) answers only.

We used the Easy-Fail trials as a baseline: if participants repeatedly failed in these trials, it indicated inattention or a general cognitive deficit. The remaining two trial types were included to examine differences in the patterns of errors participants made in their predictions, for example, to determine any performance differences in fail versus success outcomes.

To explore differences in young and older adults’ adaptability in physical reasoning, we manipulated the virtual environment gravity to investigate how participants adapted their internal representations of physical laws. For each game and trial type, we used terrestrial gravity, low gravity (half terrestrial gravity) and high gravity (double terrestrial gravity). The corresponding gravity level was written at the top of each trial, so participants were aware of the different levels of gravity but were not aware of the different trial types. Overall, participants completed 207 overall trials (23 games × 3 trial types × 3 gravities). We varied gravity at 1 g, 0.5 g, and 2 g specifically to place terrestrial physics between two equally spaced, unfamiliar yet solvable contexts, a standard manipulation for testing updating of internal physics models. Because our *a priori* hypothesis concerned adaptability to any non-terrestrial context, and to maintain reliable estimates across games and trial types, analyses collapsed 0.5 g and 2 g into a single ‘altered gravity’ level.

### 2.3 Procedure

Participants completed the experiment online from their home computers (https://www.bbk.ac.uk/psychology/e/xp/282/258/). They were first presented with an online form to obtain informed consent. This outlined that there were no known risks associated with taking part in the experiment, in addition to the experiment goals and how data would be used. Next, participants were presented with a description of the game rules and completed practice to help introduce them to the tasks. Participants were not exposed to the dynamics of the scene prior to starting the task but experienced the environment during the baseline stage of the experiment.

In each trial, participants were shown a still scene of the Virtual Tools environment which illustrated where the tool would be placed. In each trial participants were asked to judge whether, based on the type of blue tool and its placement, the red object would enter the green goal area (‘Success’) or miss it (‘Failure’) (see Figure 1). After making this choice by clicking on the corresponding button, the object movement was triggered, approximating the physics of the world, and revealing the actual outcome. The experiment was self-paced with no time limits; participants could pause *ad libitum* between trials, and Easy-Fail trials served as an attention/engagement check. The trial played until the ball stopped moving. The red ball moved across similar paths and speed profiles across the three trial types (Easy-Fail, Difficult-Fail, and Success). Trials were presented in a unique random permutation per participant (shuffle without replacement) across all game × trial-type × gravity combinations, ensuring that Easy-Fail, Difficult-Fail, Success, and gravity levels were interleaved rather than blocked. At the end of all trials, participants were presented with a debriefing page that also provided contact information for further inquiries.

### 2.4 Statistical analysis

Our primary outcome in this study was the prediction accuracy (proportion correct), computed per participant for each relevant factor combination (trial type, gravity, action concept), because it directly indexes the ability to forecast physical outcomes and therefore tests our hypotheses (H1–H3). Trials were aggregated to participant-level means; “Easy-Fail“ items served as an engagement and attention check, and single games with Easy-Fail accuracy lower than 3SD below the overall Easy-Fail mean (across participants/gravities) were excluded. This conservative rule avoids retaining items that fail to instantiate an ‘obvious fail’ baseline, thereby reducing noise and bias in group comparison. For H1 (age differences under terrestrial gravity), we first compared Easy-Fail accuracy between younger and older adults to confirm comparable engagement, then fitted a 2 (Age: younger, older) × 2 (Trial type: Difficult-Fail, Success) mixed ANOVA on terrestrial-gravity trials to test the main effect of Age and the Age × Trial-type interaction; to assess generality across stimuli we further estimated 2 (Age) × 23 (Game) and 2 (Age) × 3 (Action concept: Supporting, Launching, Clearing) ANOVAs. For H2 (adaptability to unfamiliar physics), we repeated the Easy-Fail engagement check under altered gravity (0.5 g and 2 g collapsed *a priori* to operationalise “non-terrestrial“ contexts and to stabilise estimates across 23 games), then ran a 2 (Age) × 2 (Trial type) mixed ANOVA on altered-gravity trials and a 2 (Age) × 2 (Gravity: terrestrial, altered) mixed ANOVA on overall accuracy to test the Age × Gravity interaction. For H3 (selective vulnerability by action concept), we conducted 2 (Age) × 3 (Action concept) mixed ANOVAs separately within terrestrial and altered gravity to evaluate whether age gaps scale with concept complexity (Clearing > Launching > Supporting). Analyses were performed in SPSS (Version 29).

## 3 Results

We first excluded all games in which participants did poorly in the Easy-Fail trials, indicating that these games were too difficult to solve across both age groups. The ‘Spiky’ game was the only game meeting our *a priori* threshold (see Methods) and therefore we excluded all trial types and gravities for this game across participants and age groups, Easy-Fail performance was at chance (baseline failure), indicating that this level did not meet our pre-defined reliability criterion (accuracy rate: M = 0.55, SD = 0.49).

### 3.1 Physical reasoning declines with aging

**FIGURE 2:**
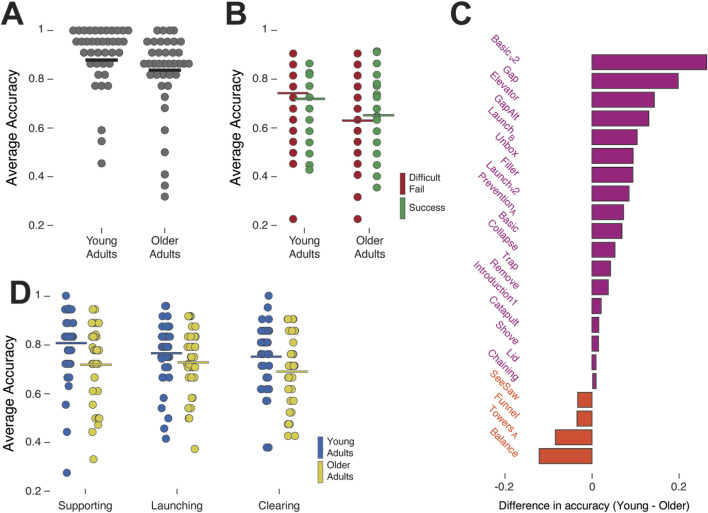
Terrestrial gravity. **(A)** Accuracy of the young and older adults in Easy-Fail trials. Each data point is the average performance of one participant in terrestrial gravity. **(B)** Accuracy in Difficult-Fail trials (red) and Success trials (green) of each one of the groups. Each data point is one participant per trial type. **(C)** Difference in accuracy between young and older adults per game. Purple bars indicate games in which younger adults showed higher mean accuracy than older adults, and orange bars indicate games in which older adults showed higher mean accuracy than younger adults. **(D)** Participants’ accuracy per action concept—Supporting (left), Launching (middle), and Clearing (right). Each data point is one participant per action concept. The accuracy is averaged across all games that include the specific action concept.

To test our first hypothesis, we focused on trials with terrestrial gravity only. We first tested age differences in Easy-Fail trials to verify that the older adults are capable of understanding the goal of the game and applying physical reasoning, are capable of spotting whether a game was not played correctly, and were attentive and played the game properly. As mentioned in the methods–the Easy-Fail trials served as a control condition, verifying that the data is reliable. Indeed, the younger and older adults performed similarly (M = 0.88, SD = 0.16, 95% CI [0.830, 0.930] and M = 0.82, SD = 0.17, 95% CI [0.767, 0.873] respectively; Figure 2A). The t-test between the two groups in the Easy-Fail shows there was no significant difference between age groups *t*(77) = 1.49, *p* = 0.13.

Next, we tested the Difficult-Fail and Success trials. A 2 (age group) × 2 (trial type) mixed ANOVA confirmed a main between-subject effect of age (*F*(1,77) = 3.57, *p* = 0.043, partial η^2^ = 0.034; Figure 2B), with younger adults performing significantly better than the older adults across both trial types (M = 0.71, SD = 0.12, 95% CI [0.672, 0.748] and M = 0.66, SD = 0.15, 95% CI [0.614, 0.706] respectively). There was no within-subject main effect of trial type (*F*(1,77) = 0.18. *p* = 0.89). We did find an interaction between age group and trial type (*F*(1,77) = 3.78, *p* = 0.048, partial η^2^ = 0.037). Sidák-corrected *post hoc* tests showed that, within younger adults, accuracy was higher on Difficult-Fail than on Success trials (Δ = 0.03, 95% CI [0.00, 0.06], Sidák *p* = 0.041; M_difficult-fail_ = 0.72, SD_difficult-fail_ = 0.14, 95% CI [0.676, 0.764] vs. M_success_ = 0.69, SD_success_ = 0.11, 95% CI [0.655, 0.725]). In contrast, older adults performed worse in ‘Difficult-Fail’ trials (Δ = 0.02, 95% CI [0.01, 0.05], Sidák *p* = 0.048; M_difficult-fail_ = 0.65, SD_difficult-fail_ = 0.15, 95% CI [0.604, 0.696] vs. M_success_ = 0.67, SD_success_ = 0.15, 95% CI [0.624, 0.716]; Figure 2B).

When comparing the two groups in the specific games, we found that the differences between age groups did not result from one specific game or a small set of games. A 2 (age group) × 23 (game) ANOVA showed a main between-subject effect of age (*F*(11,771) = 7.86, *p* = 0.005, partial *η* ^2^ = 0.004) but no main effect of game (*F*(22,1771) = 0.93, *p* = 0.54) and no Age × Game interaction (*F*(22,1771) = 1.07, *p* = 0.37). Younger adults showed numerically higher mean accuracy than older adults in 19 out of the 23 games (82.6%; Figure 2C), whereas older adults slightly higher mean accuracy in SeeSaw, Funnel, Towers-A, and Balance. Although per-game differences were small, this descriptive pattern supports the view that age-related disadvantages in physical reasoning are widespread across game types.

Finally, we tested differences between the age groups in the different physical action concepts, namely, supporting, launching, and clearing (Figure 2D). A 2 (age group) × 3 (action concept) mixed ANOVA confirmed a main between-subject effect of age (*F*(1,77) = 3.62, *p* = 0.041, partial *η* ^*2*^ = 0.035), within-subject main effect of action concept (*F*(1,77) = 6.54, *p* = 0.013, partial *η* ^*2*^ = 0.078). We also found a significant interaction between age and action concept (*F*(1,77) = 3.91, *p* = 0.038, partial *η* ^*2*^ = 0.029). Sidák-corrected *post hoc* tests showed that, collapsed across age, Clearing < Launching (Δ ≈ 0.06, 95% CI [0.01, 0.11], Sidák *p* = 0.022) and Clearing < Supporting (Δ ≈ 0.07, 95% CI [0.01, 0.12], Sidák *p* = 0.015). Simple age effects indicated that younger adults outperformed older adults in Clearing (Cohen’s d ≈ 0.45; Sidák-corrected *p* = 0.041) and also in Supporting (Cohen’s d ≈ 0.38; Sidak-corrected *p* = 0.028), whereas no reliable age difference was observed for Launching (Sidak-corrected *p* = 0.27).

### 3.2 Adapting physical reasoning is affected by aging

**FIGURE 3:**
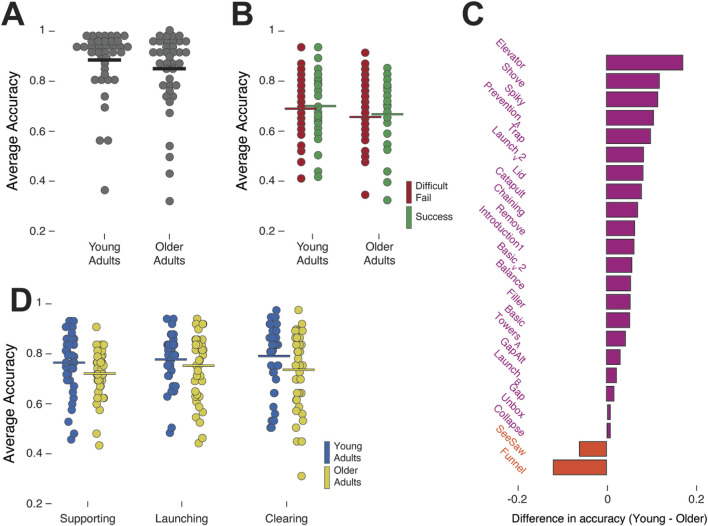
Altered gravity. **(A)** Prediction accuracy in Easy-Fail trials under altered gravity. Each data point is the average performance of one participant across half and double terrestrial gravity. **(B)** Accuracy in Difficult-Fail trials (red) and Success trials (green) of each one of the groups, average across both altered gravities. **(C)** Average difference in accuracy between young and older adults per game across the two altered gravities. Purple bars indicate games in which younger adults showed higher mean accuracy than older adults, and orange bars indicate games in which older adults showed higher mean accuracy than younger adults. **(D)** Participants’ accuracy per action concept, averaged across all games in the two altered gravities.

To test our second hypothesis, we tested trials with altered gravity only (half and double gravity combined). Similarly to terrestrial gravity, we first validated there are no age differences in Easy-Fail trials. A t-test between the two groups in the Easy-Fail trials confirmed no significant effect of age group (*M*_young_ = 0.87, *SD*_young_ = 0.13, 95% CI [0.829, 0.911] and *M*_older_ = 0.83, *SD*_older_ = 0.15, 95% CI [0.784, 0.876]; *t*(77) = 1.13, *p* = 0.26; Figure 3A).

A 2 (age group) × 2 (trial type) ANOVA on the Difficult-Fail and Success trials confirmed a main between-subject effect of age (*F*(1,77) = 3.80, *p* = 0.043, partial η^2^ = 0.037; Figure 2B) and no main within-subject effect of trial type (*F*(1,77) = 0.00, *p* = 0.99). In contrast to terrestrial gravity, there was no interaction between age group and trial type in the altered gravity trials (*F*(1,77) 0.86, *p* = 0.35; Figure 3C).

When comparing the two groups in the specific games, we found that similar to the terrestrial gravity trials, younger adults showed higher mean accuracy than older adults in most games (21 out of the 23 games (91.3%; Figure 3C) and older adults showed higher mean accuracy in the SeeSaw and Funnel games (both of them were also included in the list of games older adults were better in terrestrial gravity; Figure 2C). A 2 (age group) × 23 (game) ANOVA confirmed a main differences between age groups (*F*(11,771) = 13.95, *p* < 0.001, partial *η* ^*2*^ *=* 0.008) but no main effect of games (*F*(22,1771) = 0.63, *p* = 0.90) and no interaction (*F*(22,1771) = 0.58, *p* = 0.93).

When examining action concepts (Figure 3D), we used a 2 (age group) × 3 (action concept) mixed ANOVA to show a main between-subject effect of age (*F*(1,77) = 3.37, *p* = 0.049, partial *η* ^*2*^ = 0.042), within-subject main effect of action concept (*F*(1,77) = 7.81, *p* = 0.006, partial *η* ^*2*^ *=* 0.092). We also found a significant interaction between age and action concept *F*(1,77) = 3.54, *p* = 0.044, partial *η* ^*2*^ = 0.034. Sidák-corrected simple effects under altered gravity mirrored terrestrial results. Younger adults performed better than older adults in Clearing (Sidak-corrected *p* = 0.042) and Supporting (Sidak-corrected *p* = 0.049), with no reliable age difference for Launching (Sidak-corrected *p* = 0.29).

Finally, we tested the interaction between participants’ overall prediction accuracy across groups and gravities. The aim of this analysis was to test whether there is a link between the better prediction of younger adults compared to older adults and the requirement to adapt. A 2 (age group) × 2 (gravity) mixed ANOVA confirmed an expected main effect of age (*F*(1,77) = 3.55, *p* = 0.043, partial *η* ^*2*^ = 0.034) but no main effect of gravity (*F*(1,77) = 0.01, *p* = 0.97) or interaction between age and gravity (*F*(1,77) = 0.96, *p* = 0.32), suggesting physical reasoning declines during aging, whether adaptation is required or not.

## 4 Discussion

We examined whether aging affects physical reasoning and the adaptability of physical reasoning. Younger and older adults completed an online prediction task within the Virtual Tools framework, requiring them to foresee the outcomes of object interactions using their existing physical knowledge. To assess adaptability, we systematically altered the gravity within the task, requiring participants to adjust their internal physics models to novel gravitational conditions. As we hypothesized, younger adults outperformed older adults in predicting physical outcomes, suggesting an age-related decline in physical reasoning. Older adults did not struggle with the simpler Easy-Fail trials, however they underperformed in the more challenging Difficult-Fail and Success trials. Furthermore, although we found that altering gravity did not widen the performance gap, older adults did exhibit consistently lower accuracy than younger adults. Analyses of different *action concepts* (supporting, launching, and clearing) revealed that older adults found more complex actions, such as clearing, especially challenging. Meanwhile, a closer inspection of individual games showed that, although younger participants tended to excel in most scenarios, older adults fared comparatively better in a few tasks (e.g., SeeSaw, Funnel, Towers-A, and Balance). Collectively, these results highlight the broad nature of age-related changes in physical reasoning, while highlighting certain contexts and task demands in which the deficits are particularly pronounced—or even partially mitigated. The observed results showing overall lower accuracy in older adults are consistent with previous research (Dexter and Ossmy, 2023) that has highlighted performance indicators such as processing speed as an early marker for a general decline in cognitive control.

The lack of age-group difference in the Easy-Fail condition indicates that both younger and older adults could readily identify an obviously incorrect tool placement, and older adults were neither inattentive nor fundamentally misunderstanding the task. Thus, the observed performance deficits in the more challenging Difficult-Fail and Success trials cannot be attributed solely to a widespread decline in cognitive control that is commonly observed with increasing age, often reflected in slower processing speed and associated with changes in the frontal cortex (Dexter and Ossmy, 2023). We argue that aging impairs fine-grained physical reasoning—detecting subtle failures and forecasting precise object–object interactions—rather than basic cue use. This pattern mirrors prior evidence that older adults retain coarse physical cues but struggle with less intuitive relational inferences (e.g., mass–volume–density inversions), yielding decrements when judgments hinge on subtle contingencies (Léoni et al., 2002).

The Virtual Tools paradigm allowed us to isolate predictive reasoning from motor execution, permitting a cleaner test of age differences in intuitive physics. The lower accuracy of older adults in Difficult-Fail and Success trials therefore points to cognitive deficits in anticipating object–object interactions, over and above known age-related declines in anticipatory motor planning (Mitko and Fischer, 2020; Stöckel et al., 2017; S. Wang et al., 2020). This distinction matters practically: failures of prediction—independent of motor control—can still precipitate hazardous behaviours (e.g., misjudging trajectories or supports), contributing to falls and injuries that carry disproportionate consequences in later life (Robinovitch et al., 2013; Stevens et al., 2008). Recognising a specific reasoning-level vulnerability clarifies where interventions should act (e.g., cueing subtle failure contingencies, simplifying multi-object scenes), rather than focusing solely on motor training.

Older adults were less accurate on failure than success judgments, whereas younger adults showed the reverse pattern (Age × Trial-Type interaction). One parsimonious explanation is a positivity-biased evaluation under ambiguity: ageing is associated with a shift towards positive information (Carstensen and DeLiema, 2018), and related work indicates that such affective bias can reduce attention to negative or risk-diagnostic cues, thereby weakening detection of impending failure (Barber et al., 2020). Complementarily, age-related attentional and perceptual slowing—reduced alerting and slower visual updating—can make brief or low-salience failure cues easier to miss (e.g., Veríssimo et al., 2022; Brenner and Smeets, 2011; Kreyenmeier et al., 2022). Our pattern is consistent with this account: when cues to failure are subtle (Difficult-Fail), older adults appear more likely to adopt a success-leaning judgment policy. While we cannot adjudicate mechanism definitively here, the convergence between our behavioural asymmetry and prior demonstrations of positivity-linked underweighting of negative evidence supports a targeted design implication: environments and interfaces for older adults should amplify diagnostic failure cues and reduce multi-object ambiguity to counteract this bias.

An important question that arises from this study concerns the neural mechanisms driving age-related declines in physical reasoning. Situations that require forward simulation of multiple potential outcomes—particularly ambiguous failures—have been shown to recruit frontoparietal network (FPN) circuitry during physical inference (Pramod et al., 2022; Zbären et al., 2023). While our Difficult-Fail trials are behavioural and not identical to those paradigms, they share the same computational demands (multi-object, branching interactions), so the observed age deficit is consistent with reduced efficiency in FPN-supported simulation—an interpretation that should be tested directly with neuroimaging using this task. More broadly, the FPN is implicated in forward-modelled object dynamics (Fischer and Mahon, 2021; Heckner et al., 2023), and precise prediction benefits from continuous visual updating yet degrades when acceleration changes must be integrated (Brenner and Smeets, 2011; Kreyenmeier et al., 2022). Age-related reductions in FPN connectivity (Oschmann and Gawryluk, 2020) therefore offer a parsimonious neural account that aligns with our behavioural asymmetry: older adults were selectively less accurate precisely when multi-trajectory simulation is required (Difficult-Fail). We present this as a targeted, testable hypothesis for future neuroimaging work using the current task.

Contrary to expectations, adapting to half or double gravity did not significantly exacerbate the performance gap between younger and older adults. Although older adults’ accuracy remained lower overall, they appeared equally challenged across terrestrial and non-terrestrial gravities, suggesting that the core difficulty stemmed from general prediction demands rather than the need to recalibrate to a novel gravitational context. Despite the recognized importance of adaptability in everyday life (Synofzik et al., 2006) and in future challenges of humankind such as climate change or space travel (Gravano et al., 2021; Grothmann and Patt, 2005; Verchick, 2016), little work has directly explored how aging impacts the recalibration of physics models under novel or extreme conditions (Smith et al., 2013). Our data address this gap by showing that while aging negatively affects physical reasoning across the board, it does not selectively impair individuals’ capacity to adapt to new gravity settings, at least under the circumstances tested here. Practically, this shifts the focus from generic ‘recalibration training’ to interventions that reduce local, multi-object complexity and amplify diagnostic cues—principles directly relevant to fall-prevention, road/transport interfaces, and VR/AR or teleoperation design. More broadly, the result identifies a resilient cognitive asset in ageing—maintained, updateable priors over global physics—that can be leveraged in assistive technologies and re-skilling contexts to support autonomy and safe mobility. Thus, future investigations could systematically manipulate other physical parameters, incorporate feedback-based training, or extend practice sessions to determine whether older adults might indeed exhibit slower—but ultimately successful—adaptation with targeted support (Mrowca et al., 2018; Smith and Vul, 2013).

Participants’ performance in the three action concepts—supporting, launching, and clearing—probe which forms of physical reasoning might be most vulnerable to age-related decline. Although older adults underperformed overall, they struggled the most with clearing (Figure 2D), which involves coordinating multiple object movements. Previous developmental research has found that children’s performances across these different action concepts are in line with the emergence of corresponding motor skills. The parallel between the order of physical action concepts by which physical reasoning emerges in early childhood, and declines in late adulthood, suggests that motor skills requiring prediction about clearing may be the first to degrade in older adulthood. In addition, older adults’ prediction in the launching games, which remained relatively robust, resonates with prior evidence that simpler, more direct trajectories are easier to anticipate (Léoni et al., 2002). However, because these data are cross-sectional, we cannot infer within-person decline, only that age was selectively associated with these differences in performance. Future longitudinal and reliability studies should test whether Clearing and Launching tracks change within individuals and can serve as an early screening target for intervention. Moreover, because participants merely observed the tools rather than actively manipulating them, further studies should incorporate first-person, embodied perspectives to confirm whether the decline in advanced action-concept reasoning remains consistent across various task formats.

As our study was done online, it has few important limitation. First, it did not include formal screening of global cognitive status or visual acuity, and participant hardware (display size, resolution, refresh rates) was uncontrolled, which may influence the perception of subtle multi-object interactions. We also did not administer formal cognitive status, educational background, or computer-experience measures, nor standardised vision tests. Although Easy-Fail trials and a fully randomised, interleaved order helped monitor engagement, they cannot preclude residual effects of attention, vision, or device variability. Our binary response measure (accuracy) also limits inferences about response criteria, and the cross-sectional design cannot establish within-person change. Future studies should incorporate brief cognition/vision screens, device calibration or laboratory replication with standardised displays, and richer measures (e.g., confidence/RT) to strengthen mechanistic interpretation.

Moreover, we argue that future research should focus on exploring any age differences in the use of physical simulations to inform decisions about how the physical world unfolds. Our results isolate two priorities for future work. First, to test mechanisms, neuroimaging with the present task—especially Difficult-Fail and Clearing trials—should assess whether age differences track efficiency within frontoparietal simulation networks and whether this predicts behaviour. Second, to improve performance, interventions should target the locus of deficit we observed—complex, multi-object contingencies—by simplifying interaction structure and enhancing early failure cues, rather than training generic recalibration (which appeared relatively preserved across gravity changes). Longitudinal and test–retest studies should evaluate whether Clearing provides a sensitive behavioural marker of age-related change and whether the aforementioned design principles translate into safer interfaces and environments for older adults.

Finally, we suggest that understanding how physical reasoning varies with age can inform—and be informed by—artificial intelligence. Similar to previous studies testing generalisation across the lifespan (Bernal et al., 2024; Ossmy et al., 2024; Ossmy and Mukamel, 2017; Mrowca et al., 2018), insights into how older adults adapt (or fail to) in complex, multi-object contexts can inspire AI systems that learn and generalise incrementally rather than from static training. Conversely, AI models and controlled simulations can help identify which computations are most vulnerable in ageing and test targeted interventions that support everyday physical reasoning. Ultimately, such integrative efforts promise not only to advance basic science but also to yield practical applications that enhance health, safety, and independence for older adults.

Taken together, our findings suggest that older adults maintain baseline intuitive physics, but increasing interactional complexity—especially multi-object contingencies—imposes greater cognitive load and reveals a selective decline in predictive reasoning independent of motor execution. This dissociation identifies concrete levers for training and design (reducing interactional complexity, amplifying diagnostic failure cues) to bolster predictive control and reduce fall risk in everyday settings.

## Data Availability

The datasets presented in this study can be found in online repositories. The names of the repository/repositories and accession number(s) can be found below: https://github.com/Physical-Cognition-Lab/Reasoning-decline-during-aging.
