# Supplementary material for: Reasoning decline during aging under familiar and unfamiliar physics
Source: Front Aging. 2025 Dec 16;6:1646655. doi: 10.3389/fragi.2025.1646655 (PMC12748166; doi:10.3389/fragi.2025.1646655)
Supplement: Supplementary file 1 [file Table1.docx]

**Supplementary Materials**

**Table S1**

Virtual Tool scenes and mapping to physical action concepts

| Virtual Tools Scene | Physical Action Concepts |
| --- | --- |
| Balance | supporting |
| Basic | launching |
| Basic_v2 | launching |
| Catapult | launching |
| Chaining | launching |
| Collapse | clearing |
| Elevator | supporting |
| Filler | supporting |
| Funnel | launching |
| Gap | supporting |
| GapAlt | supporting |
| Introduction1 | supporting |
| Launch_B | launching |
| Launch_v2 | launching |
| Lid | clearing |
| Prevention_A | clearing |
| Remove | clearing |
| SeeSaw | supporting |
| Shove | clearing |
| Spiky | supporting |
| Towers_A | launching |
| Trap | clearing |
| Unbox | clearing |

*Note.* List of all virtual tools scenes we used in this study and their mapping to physical action concepts (See more information in Grandchamp des Raux et al., 2024).
